# Supplementary material for: Label-free characterization of organic nanocarriers reveals persistent single molecule cores for hydrocarbon sequestration
Source: Nat Commun. 2021 May 25;12:3123. doi: 10.1038/s41467-021-23382-8 (PMC8149835; doi:10.1038/s41467-021-23382-8)
Supplement: Supplementary file 1 — Supplementary Information [file 41467_2021_23382_MOESM1_ESM.pdf]

Supplementary Information For

Label-free characterization of organic nanocarriers reveals persistent single molecule cores for hydrocarbon sequestration

Terry McAfee[1,2], Thomas Ferron[1], Isvar A. Cordova[2], Phillip D. Pickett[3], Charles L. McCormick[3], Cheng Wang[2*], Brian A. Collins[1*]
Correspondence to: brian.collins@wsu.edu; cwang2@lbl.gov

Supplementary Methods

RSoXS Quantitative Spatiochemical Analysis

Overall Scattering Model: Reduced 1-dimensional RSOXS profiles $\left(I(\mathbf{q}, E)\right)$ were fit to a combined scattering model of a spherical polydisperse core-shell form factor $\left(P_{\mathrm{CS}}(\mathbf{q}, E)\right)$ and a Percus-Yevick hard-sphere structure factor $\left(S_{\mathrm{HS}}(\mathbf{q})\right)$ through the equation:

$$I(\mathbf{q}, \mathrm{E}) = A \cdot T(E) \cdot P_{\mathrm{cs}}(\mathbf{q}, E) \cdot \mathrm{S}_{\mathrm{HS}}(\mathbf{q}) + I_{\mathrm{bkg}}(\mathbf{q}, E)$$

where $\boldsymbol{q}$ is the momentum transfer vector, E is the photon energy, A is a global scale parameter, $T(E)$ is an attenuation parameter, and $I_{\mathrm{bkg}}(\mathbf{q}, E)$ is an energy dependent background. The use of a global scale parameter is due to RSoXS not being calibrated for an absolute intensity.

Form Factor: The form factor $P_{\mathrm{cs}}(\mathbf{q}, E)$ utilized in this study describes an inner spherical core of radius r_c surrounded by a constant shell of thickness r_{sh} with complex index of refractions given by n_c and n_{sh} respectively. In order to account for polydispersity in the particle size, we modify the core-shell model with a given core radius r $\left(F_{\mathrm{CS}}(r, \mathbf{q}, E)\right)$ with a Schultz-Zimm distribution through the equation:

$$P_{\mathrm{cs}}(\mathbf{q}, E) = \int_0^{\infty} \mathcal{P}(r_{\mathrm{c}}, \sigma_{\mathrm{c}}) \frac{|F_{\mathrm{cs}}(r, \mathbf{q}, E)|^2}{\bar{V}(r + r_{\mathrm{s}})} \mathrm{dr}$$

Where $\mathcal{P}(r_c, \sigma_c)$ is the Schultz-Zimm probability density of average radius r_{c} with width σ_{c} and $\bar{V}(r)$ is the average volume of a sphere between radii r and $r + \mathrm{dr}$. Fit functions that included polydisperse shell thickness were explored to better describe potential particle effects but were ultimately removed due to a negligible impact on fit results while increasing parameter space. The individual form factor is given by the following function developed by Guinier and Fournet.(*1*)

$$F_{\mathrm{cs}}(r, \mathbf{q}, E) = V(r)\Delta n_{c,s}(\mathrm{E})\Phi(\mathbf{q} \cdot r) + V(r + r_{\mathrm{s}})\Delta n_{\mathrm{s,sol}}(E)\Phi\big(\mathbf{q}(r + r_{\mathrm{s}})\big)$$

Here $V(r)$ is the volume of a sphere with radius r, $\Delta n_{i,j}$ is the difference in complex index of refraction between domain i and j (with subscripts c, sh, and sol referencing the core, shell, and solvent), and $\Phi(\mathbf{q} \cdot r)$ is the scattering form factor of a sphere with radius r given by:

$$\Phi(x) = \frac{3[\sin(x) - x\cos(x)]}{x^3}$$

Altogether, the form factor contributes three structural parameters: average core radius, r_c, width of core distribution, σ_c, and shell thickness r_s. Chemical identity is incorporated into the form factor through the energy-dependent difference in complex index, where $\Delta \mathrm{n}_{\mathrm{ij}}(E) = \Delta\delta_{\mathrm{ij}}(E) + \Delta\beta_{\mathrm{ij}}(E)$.

Structure Factor: Interparticle correlations are described by a hard-sphere structure factor, $S_{\mathrm{HS}}(\mathbf{q})$, given by the Percus-Yevick equation.(*2*) This approximation adds two independent fit parameters, R_{HS} gives the impenetrable radius of a single particle and ϕ_{HS} gives the volume fraction occupied by the particle population.

Attenuation: The attenuation of the beam $T(E)$ was modeled from the Beer-Lambert law and is given by:

$$T(E) = 1 - e^{-\frac{4\pi E}{hc}\alpha \sum_i \phi_i \beta_i(E)}$$

where h is Planks constant, c is the speed of light, α is the areal density, and ϕ_i is the volume fraction for the ith domain. The volume fraction occupied by micelles is held to the structure parameter ϕ_{HS} and α is an open parameter. $\beta(E)$ is constructed for the micelle domain as a weighted sum of the measured optical constants based on the average chemical composition.

Background: Potential scattering backgrounds are described through a power-law:

$$I_{bkg}(\mathbf{q}, E) = B(E)q^{p(E)} + C(E)$$

where $B(E)$ and $C(E)$ are energy-dependent backgrounds and $p(E)$ is a power law. We note, setting $B = 0$ does not impact the quality or results of some energy fits if the scattering power is large. The power-law background primarily accounts for the upturn in low-Q scattering for non-resonant energies and does not interfere with other model parameters.

Fitting Procedure: In addition to the structural parameters, the analysis incorporates optical parameters (δ, β) for each portion of the structure. Because significant hydration was expected in the shell, the fitting was initiated with optical parameters of a pure PPO core and pure H_2O shell based on our NEXAFS measurements. Since the desired output of the analysis was the shell chemical composition, the shell optical parameters were the first focus of refinement. Due to correlations between δ and β, only one parameter was allowed to optimize at a time, alternating until an optimized structure was found. Subsequently, the core optical parameters were opened to further improve the result. This required only slight refinements from our measured optical constants to optimize the fit. This is most likely due to density variations in the micelle and uncertainty with our independent NEXAFS measurements. To confirm the final optical parameters of the shell as well as estimate uncertainties in the final values, we reran the fitting procedure starting from a 50% PEO shell.

All final fit parameters are given in Supplementary table 2 (Energy independent) and Supplementary table 3 (Energy dependent) and a correlation matrix is given for the structural parameters in Supplementary figure 1.

Determining the Chemical Composition of the Corona

To determine the chemical composition of the micelle corona we modeled the contrast functions $|\Delta n_{c,sh}(E)|^2$ and $|\Delta n_{sh,sol}(E)|^2$ with the following parameterization:

$$n_c = n_{PPO}$$
$$n_{sh} = \phi_{PEO} n_{PEO} + \phi_{H_2O} n_{H_2O}$$
$$n_{sol} = n_{H_2O}$$

Where ϕ_x is the volume fraction of the xth component in the corona with $\phi_{PE} + \phi_{H_2O} = 1$. Since RSoXS was not calibrated on an absolute intensity we approximated the core to be composed of 100% PPO and the solvent to be 100% H_2O. The impact of shell hydration on both contrast functions is demonstrated in Supplementary figure 2. Note how the contrast between the shell and solvent drop over five orders of magnitude as the concentration of PEO within the shell tends to zero. It is this variability in the expected contrast (and subsequently scattering intensity) that allows us to determine the composition of the shell.

The ratio between $|\Delta n_{c,sh}|^2$ and $|\Delta n_{sh,sol}|^2$ is calculated from the fit parameters and finds that up to three orders of magnitude more scattering is expected from the core – shell interaction compared to the shell – solvent. A composition of the shell is calculated by fitting the contrast ratio from the RSoXS profiles (see previous section) to the calculated ratio from Supplementary figure 3 with ϕ_{PEO} as a fit parameter. The

uncertainty in the ratio is from the parameter uncertainty (calculated from the fitting algorithm) and the fluctuation in the X-ray beam intensity (as shown in Supplementary figure 4) added in quadrature.

Geometric calculations of core and shell radii and shell hydration

The amount of water present in the pluronic F127 micelle shell can be estimated independent of the optical constants by using the radii extracted from the fits in Figure 2a if we assume the core is pure PPO, and there is no PPO in the shell, which are good assumptions based on both the DPD simulations and the literature. The number of polymer chains in the micelle can be calculated by knowing the core radius, the density of PPO = 1.04 g per cm^3, and that there are 65 PO monomers per polymer chain. Since the ratio of EO monomers to PO monomers is 200:65, and the density of PEO is 1.12 g per cm^3, the volume occupied by PEO can then be easily calculated using and compared to the shell volume.

Assuming the density of DDAM is the same as AMPS, 1.1 g per cm^3, the volume of a single molecule would occupy ~21.4 nm^3 if it forms a solid, non-hydrated sphere. From our study of F127, as well as literature reports, the hydrophilic component is likely heavily solvated, while the hydrophobic portion is not. If the hydrophobic DDAM portion is assumed to be pure, but the hydrophilic AMPS portion is 90% water, a pure DDAM core would have a volume of 9.4 nm^3 for and a hydrated AMPS shell volume would be 210 nm^3. This results in a core radius of 1.31 nm and total radius of 3.74 nm. However, due to the statistical nature of the copolymer, it is likely that the core also incorporates significant hydrophilic AMPS monomers and even possibly a small hydration sphere, swelling the core significantly. This is consistent with the larger core radius measured in this work.

Dissipative Particle Dynamics simulation

The illustration in Figure 2e of a self-assembled micelle structure was the result of Dissipative Particle Dynamics (DPD) simulations, a coarse grain, bead-based, simulation method that was performed using the Mesocite module in Materials Studio 5. The simulation parameters, including the conservative force parameters shown in the table below, are based on those previously published.(*3*) Each water bead represents three water molecules, setting the bead mass to 54 amu and length scale to 6.46 Å. Each EO bead represents 4 EO monomer units, each PO bead represents 3 PO monomer unts, allowing F127 to be modeled by 23 EO beads - 21 PO beads - 23 EO beads. A time step of 254 fs and energy scale of 0.5922 kcal/mol were used. The interaction between beads is governed by the conservative force parameters shown in Supplementary table 1.

F127 Concentration dependence

As we monitor the replacement of the H_2O in the cell with 1 wt. % F127, since only the volume fraction is of interest in this experiment, the form factor model used for these fits was simplified from a core-shell structure to just a sphere, with a Shulz-Zimm distribution. This model fit well to a radius of 14.3 nm, a distribution width of 6.1 nm, and a hard sphere structure factor radius of 9.3 nm. A broad, dilute population with a Shulz-Zimm distribution was also fit to the model to account for low-q scattering from aggregates in the un-filtered solution. All the profiles in Figure 3a were fit to these same two distributions, over the same data range. Only the volume fraction and overall intensities were allowed to change between traces.

The volume fraction parameter from the profile fits was plotted verses time in Figure 3b and the trend was itself fit using a double exponential function: $f(t) = A - B * \mathrm{e}^{\frac{-(t-t_0)}{\tau_1}} - C * \mathrm{e}^{\frac{-(t-t_0)}{\tau_2}}$, with A being the

maximum volume fraction of the micelles. The resulting fit yields: $A = 0.26(1)$, $B = 0.143(7)$, $C = 0.105(9)$, and time constants $\tau_1 = 0.57(6)$ min, and $\tau_2 = 13(4)$ min. The larger time constant, τ_2, is related to the path length of tubing between the syringe pump distributing the F127, and the entrance to the flow cell. We hypothesize that τ_1 is related to a combination of mixing within the small dead volume inside of the cell, as well as the sample having to travel ~2mm between the two silicon nitride membranes, spaced only 500 nm apart, to reach the measurement region within the cell.

aPS50 Concentration Dynamics

The RSoXS data shown in Figure 4c was collected and fit using the same procedure used for F127 in Figure 3a, as described above. The photon energy used was 287.3eV, which maximized the contrast between the DDAM and AMPS monomers within the chain so as to enhance the core-shell contrast, resulting in the highest signal-to-noise of any energy. This can be seen the spectral analysis shown in Supplementary figure 7 and the variation of scattering patterns displayed in Supplementary figure 8. Since there was a clear change in the location of the scattering feature between the two micelle systems, the form factor and structure factor were also allowed to vary in the fit. The volume fraction vs concentration extracted from the fits are shown in Supplementary figure 9.

To calibrate the real-time concentration during the dynamics experiment, static control concentrations were flowed in the instrument and measured. The scattering peak positions from these static controls were matched with those of the dynamic transition experiment to determine the concentration at specific flow times. A polynomial spline was assessed to this data and used to convert the remaining flow times into concentrations.

Synthesis of aPS50

The general procedure is as follows. The DDAM and AMPS with desired feed ratio, for example, DDAM (0.9717 g, 3 mmol), AMPS (0.6224 g, 3 mmol), CPDT (22.6 mg, 0.06 mmol), DMF (3 mL) and AIBN (4.9 mg, 0.03 mmol) with the molar ratios of DDAM: AMPS: DMP: AIBN= 100: 100: 2: 1, were added to a 10 mL polymerization flask equipped with a magnetic stir bar. After purging with N_2 for 30 minutes, the flask was placed in an oil bath at 70 °C while stirring. After a desired polymerization time, the flask was removed from the oil bath and immediately cooled with liquid nitrogen and opened to atmospheric pressure. The crude reaction solution was then dialyzed against methanol three times over 1 day followed by water with small amount of HCL three times over another day. The purified product was isolated by lyophilization resulting in a fine, white powder. This sample (called aPS50 in the manuscript) contains 50 mole % DDAM monomer along its backbone and has a total weight average molecular mass (Mw) of 14.2 kDa (Đ = 1.2). Structural data for the aPS50 polymer was collected using ^{1}H-NMR for determining %DDAM content and gel permeation chromatography for molecular weight and PDI information.

Pyrene Uptake of aPS50

To demonstrate that aPS50 serves as a hydrocarbon sequestering micelle/nanocarrier, we evaluate its ability to uptake pyrene into its core domain. The following procedure was used to load pyrene into aPS50 micelles. 5 µL of 50 mg per mL pyrene solution in acetone was added to each 0.5 mL well of a 96-well plate. After all of acetone was evaporated, 0.5 mL of aPS50 in DI water with the desired concentration was added to the respective well to solubilize the pyrene deposited on the surface. The contents were allowed to equilibrate with shaking over a one day period. Afterwards, a 0.2 mL aliquot of aPS50 solution was removed and transferred to a new 96-well plate and the amount of pyrene in solution was determined by UV-Vis absorbance spectroscopy at a wavelength of 341 nm. Fluorescence spectroscopy was also used to probe the hydrophobic domain formation of the micelles via the I_3/I_1 ratio of pyrene in solution.

Supplementary Discussion

Sample Integrity Considerations

Beam damage and interactions of the sample with the measurement cell must be considered and evaluated for each material system investigated by this new in-situ RSoXS technique. Beam damage was investigated by performing sequential measurements on a static sample and observing the scattering pattern. Although beam damage was not observed for the doses used to collect a complete set of scattering data at multiple energies, dynamic investigations require significantly higher total dosage. To avoid cumulative beam damage effects, the sample is flowed through the cell at a constant rate during measurements.

When flowing a sample through the measurement cell, it is critical to ensure that flowing the sample does not change its morphology. Thus, a comparison of the sample in static conditions and under flow conditions must be performed. For aPS50, a comparison between the sample in static conditions was compared to the sample flowing at the measurement conditions of 50 µL per hour, and no difference was observed. When using a 500 nm spacer, which was used for all data included in this work, F127 was found to have no observable changes to the scattering pattern when comparing static conditions with flow rates of 50, 100, 200 and 300 µL per hour. This range was selected because 300 µL per hour is the maximum recommended flowrate for the cell, and 50 µL per hour is the flowrate we use during in-situ measurements to minimize the total amount of sample needed. However, a spacer thickness of 150 nm was used during early investigations of F127 and it was found that micelles did not form under any flow conditions. This was surprising to the authors since the micelle is nominally only ~25 nm in diameter, 6 times smaller than the channel width. This indicates there may be an interaction of the micelles with the Silicon Nitride that provides the barrier between vacuum and the measurement volume. If interaction does occur it is possible that the narrower channel introduces shear induced dissociation of the micelles.

The last cleaning step for the e-chips is UV-ozone or Plasma cleaning, which in addition to removing any remaining residue on the surface, it makes the Silicon Nitride surface hydrophilic. Having a hydrophilic surface for aqueous samples makes it considerably easier to assemble the flow cell correctly, which saves valuable experiment time and reduces the amount of broken Silicon Nitride windows. The downside is that aqueous micelles tend to have a hydrophilic component, which means they will be attracted to the Silicon nitride window as well. Thus, it is critical to evaluate if and how your sample interacts with the measurement cell. From figure 3b in the main text, F127 is observed to have a volume fraction of over 20%, despite the sample being only 1 wt. % F127. This indicates there is an enrichment of micelles within the flow cell. The observed enrichment coupled with the fact that flowing through a 150 nm channel destroys the micelles leads to the conclusion that the F127 molecules are attracted to the Silicon nitride membrane. We found that the enrichment of F127 can be reduced substantially by skipping the UV-Ozone cleaning. For this work, we utilized the enrichment to allow fitting the structure factor component, and contrast the evolution of the structure factor with what is observed for aPS50. After in-situ experiments were complete, the cell is purged with water to see if there is any residual signal from the sample. For F127, there is always some residue stuck to the Silicon nitride window, however the signal intensity from this is negligible compared to the signal intensity when the sample is present. For aPS50, we did not observe any residue stuck to the window after measurement, nor any other evidence of the aPS50 interacting with the Silicon nitride membrane.

Supplementary Figures.

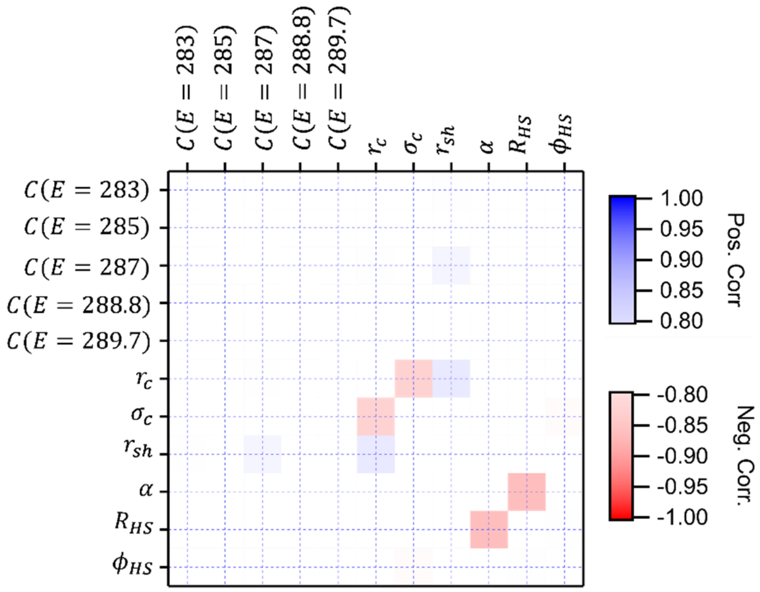

Supplementary figure 1.
Correlation matrix for structural parameters in multi-energy F127 fitting. The highest correlation between α and R_{HS} was found to be 0.86. All energy-dependent parameters were held during calculation.

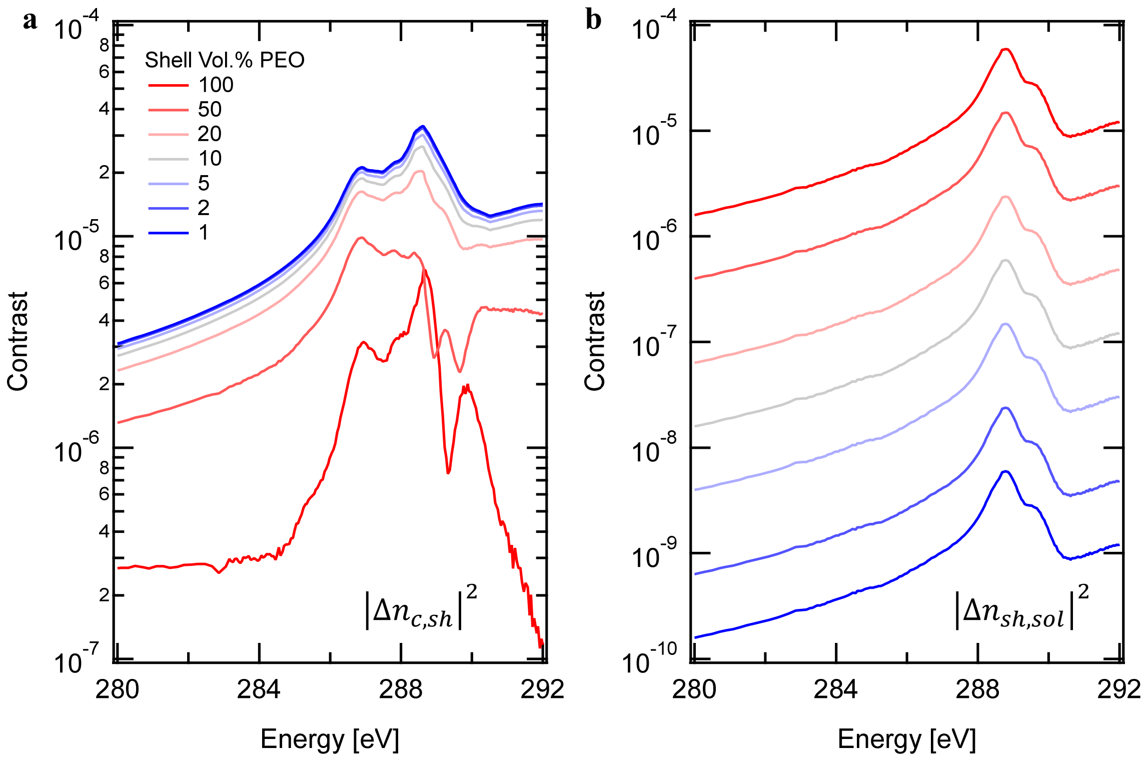

Supplementary figure 2.
Calculated scattering contrast between the (a) core-shell and (b) shell-solvent interactions with varying PE volume fraction in the shell. Color scale is identical for all subgraphs.

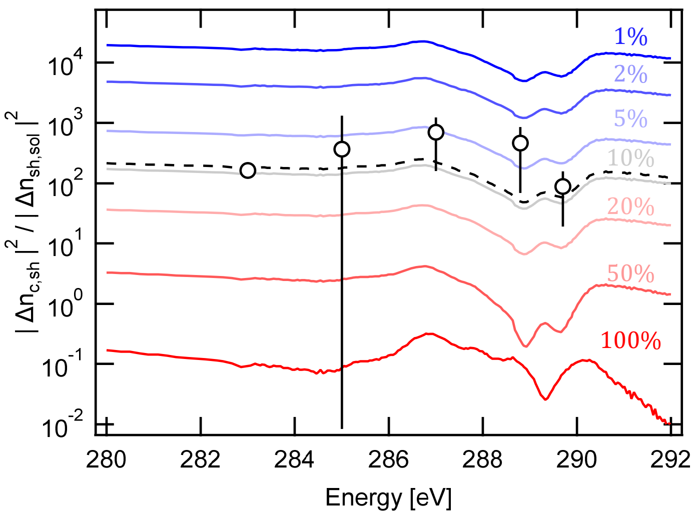

Supplementary figure 3.
Ratio between the contrast calculated in Supplementary figure 2. PE Vol.% in the shell is indicated within the figure. Open circles represent fit parameters from the multi-energy fit in Figure 2a and Supporting Table 3. Dashed line gives the best fit to the measured data.

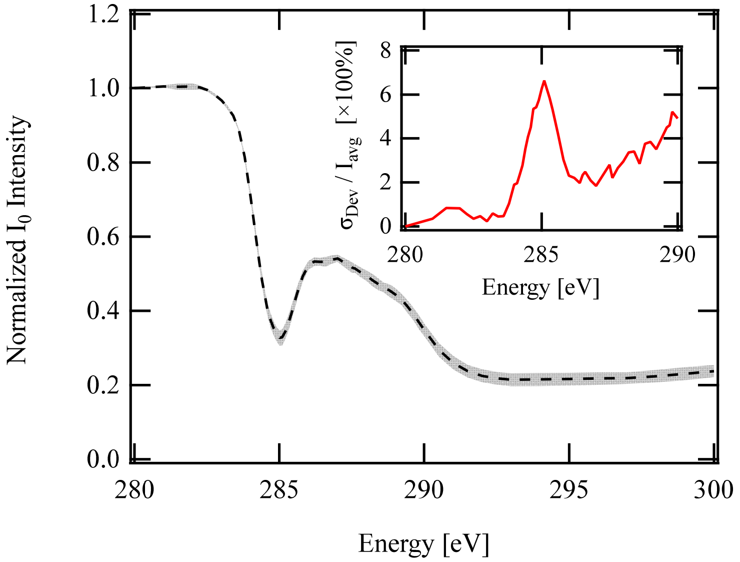

Supplementary figure 4.
Normalized direct beam intensity at RSoXS instrument. Grey color bands represent standard deviation of I_0 monitoring during data collection. Inset graph highlights σ_{Dev}/I_{avg} in energy range of interest and acts as additional uncertainty in energy space uncertainty parameters.

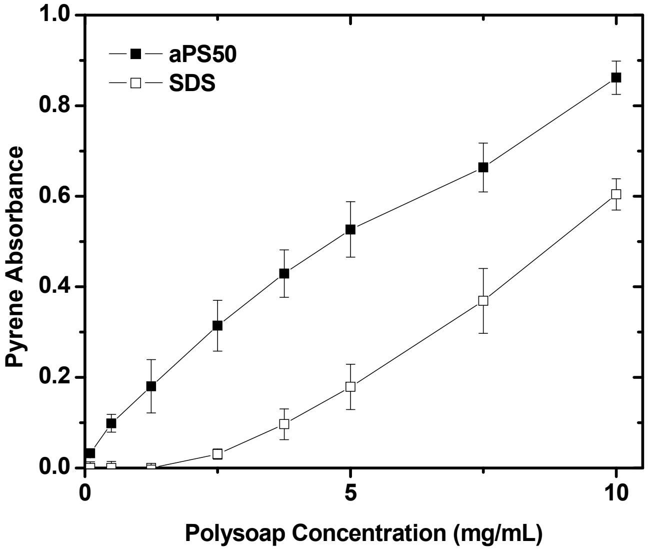

Supplementary figure 5.
Pyrene sequestration as measured by UV absorbance at 341nm as a function of aPS50 concentration. SDS is used as a control. The pyrene absorbance increases linearly with polymer concentration in accordance with Beers Law. SDS displays a CMC, evident by zero pyrene absorbance at 2.5 mg/mL. However, aPS50 does not exhibit a measurable CMC and is more efficient at pyrene sequestration with greater absorption values when compared to SDS.

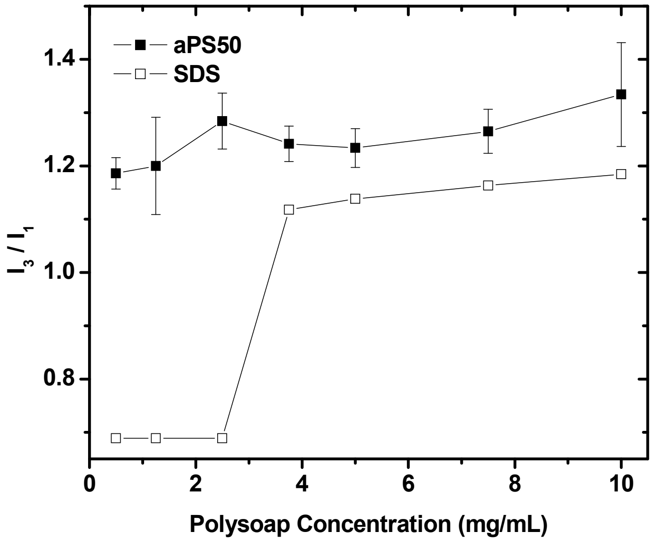

Supplementary figure 6.
Fluorescence spectrum I_3/I_1 values vs aPS50 concentration in aqueous solution with pyrene as a probe. SDS is used as a control. Excitation wavelength was 341nm. Below the CMC of SDS, pyrene has an I_3/I_1 ratio of 0.68 in water and above the CMC the ratio increases to 1.1. The aPS50 sample exhibits elevated ratios ($I_3/I_1 > 1.1$) throughout the entire concentration range probed. This indicates significant hydrophobic domain formation even at dilute concentrations.

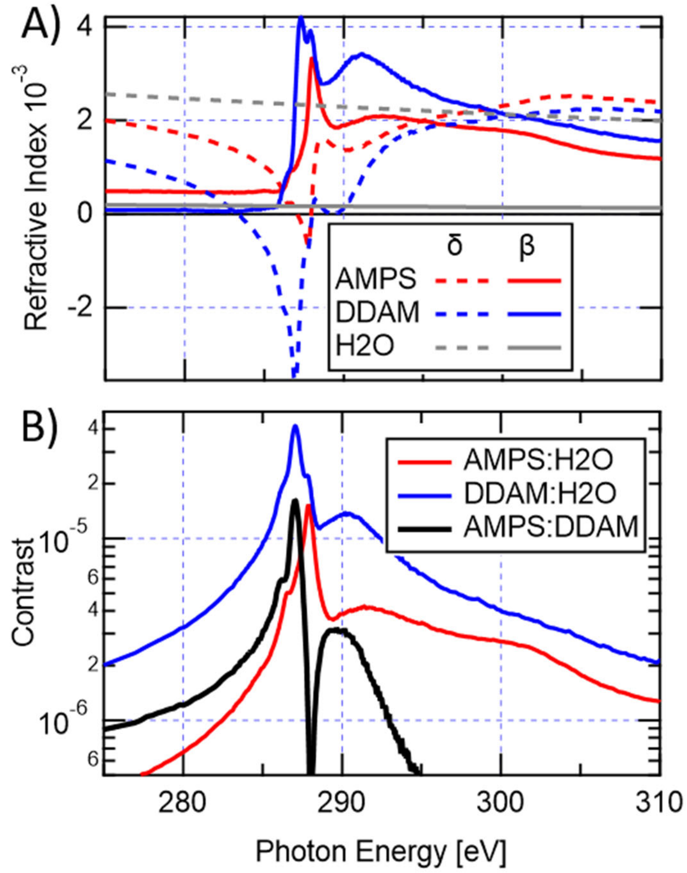

Supplementary figure 7.
A) Optical parameters for AMPS and DDAM monomers compared with water. Data from NEXAFS spectroscopy via total electron yield measurements of drop-cast films. Data acquired at beamline 11.0.1.2 of the ALS. Density for DDAM and AMPS are both assumed to be 1.1 g per cm^3. B) Scattering contrast $|\Delta n|^2$ from data in A) calculated between the three components within the solution demonstrating maximum scattering contrast at the resonance at a photon energy of 287.3 eV used in the study.

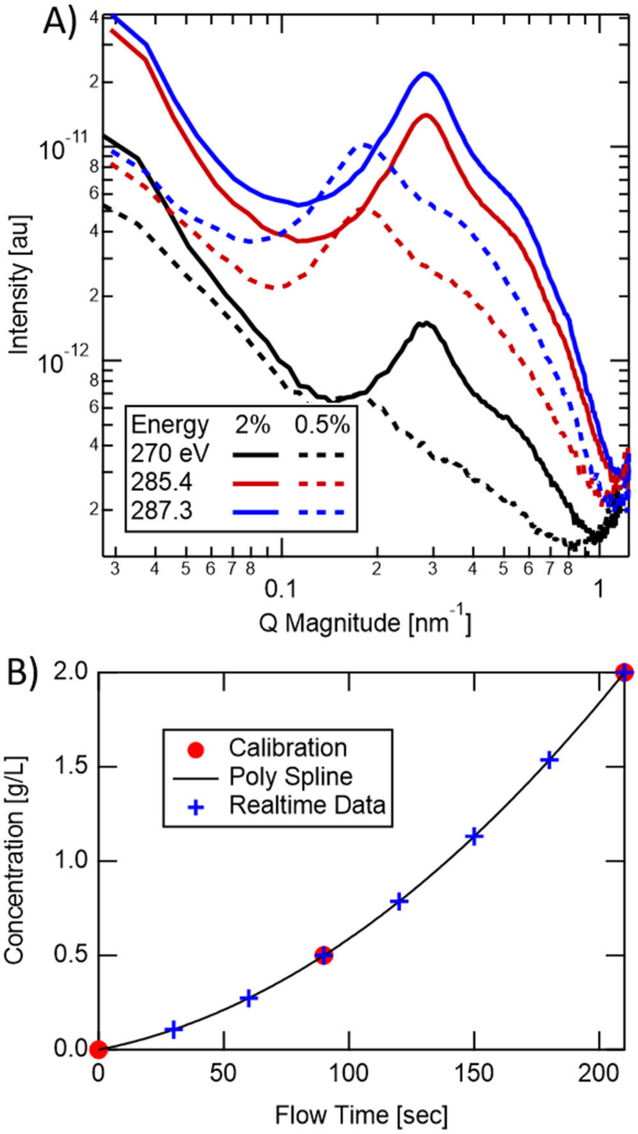

Supplementary figure 8.
A) RSoXS scattering profiles for static concentrations of aPS50 at 0.5 and 2 wt.% in water. The energy dependence on of the scattering profiles is also apparent and follows the calculated contrast functions for this system. The shifted peaks correlate with that seen in the dynamic study (Figure 4), indicating all measurements are at quasi equilibrium rather than a non-equilibrium structure caused by the concentration gradient. B) Calibration of the real-time data for concentration. Red circles are from the calibration data in A) by matching scattering peak positions of the static samples (known concentration) versus the dynamic ones (known flow time). The real-time data was then calibrated to the fit polynomial spline.

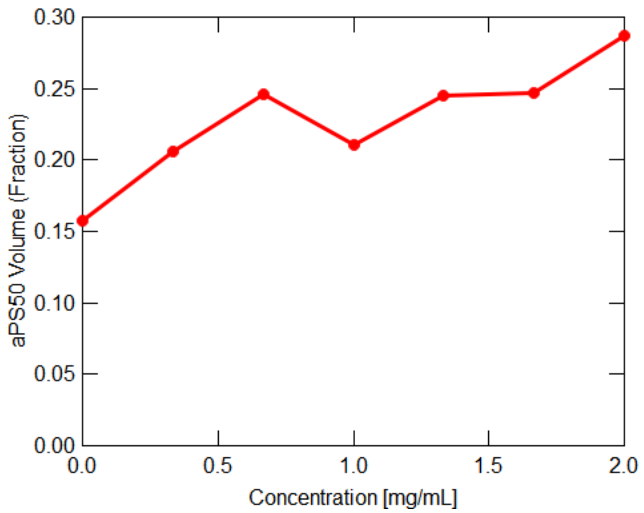

Supplementary figure 9.
Volume fraction verses concentration for aPS50 extracted from the fits in Figure 4c Even in the very dilute sample, the volume fraction indicates there is significant interaction between micelles, which supports the DLS data that indicates there are always larger clusters of many micelles.

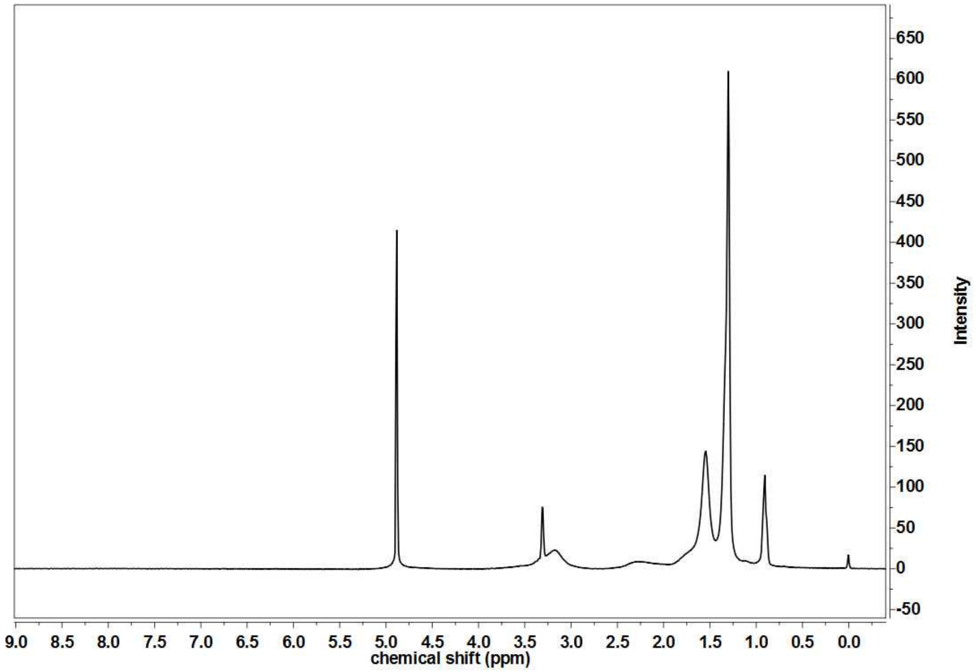

Supplementary figure 10.
^{1}H-NMR of polysoap aPS50 in Methanol-d_4.

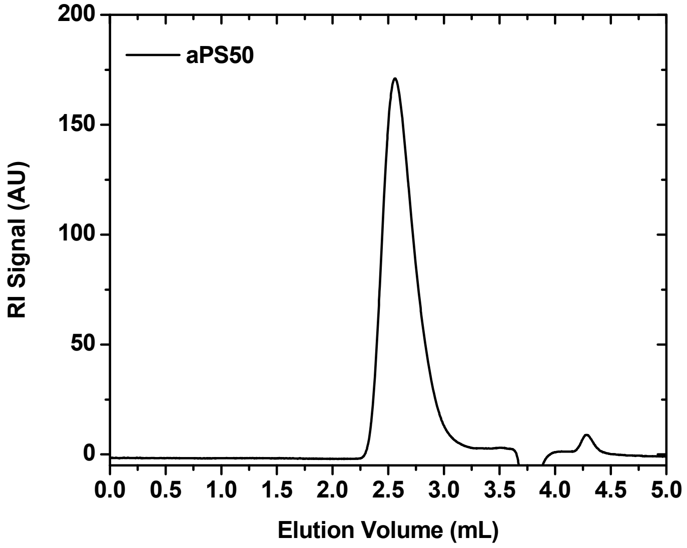

Supplementary figure 11.
GPC trace of polysoap aPS50 in 0.2M $LiClO_4$ in methanol at a flow rate of 0.6 ml/min.

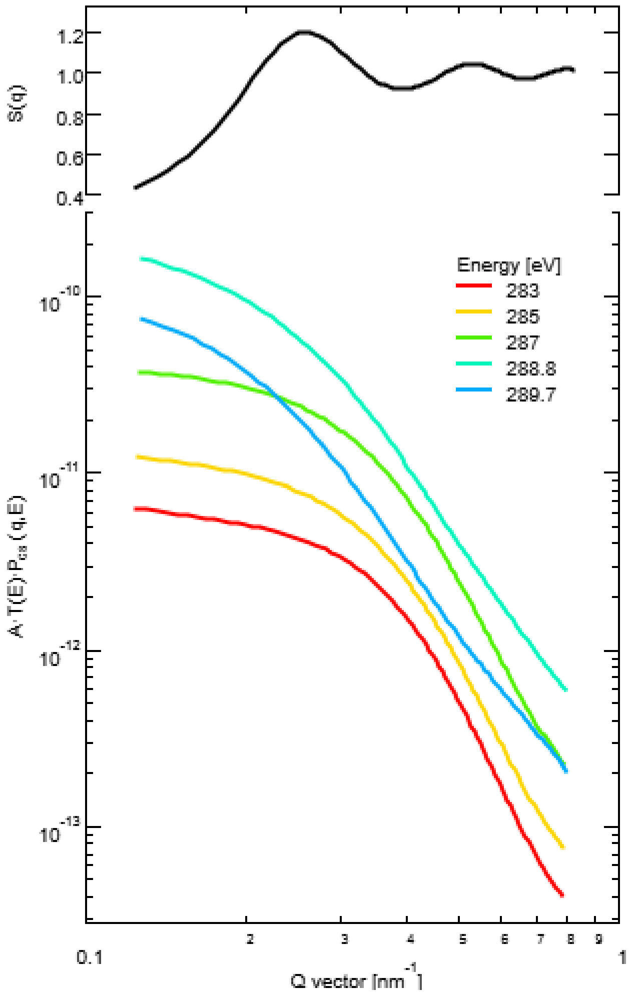

Supplementary figure 12.
Structure Factor (top) and Form Factor (bottom) components for figure 2a. All energies have the same Structure Factor, but the Form Factor varies significantly with energy.

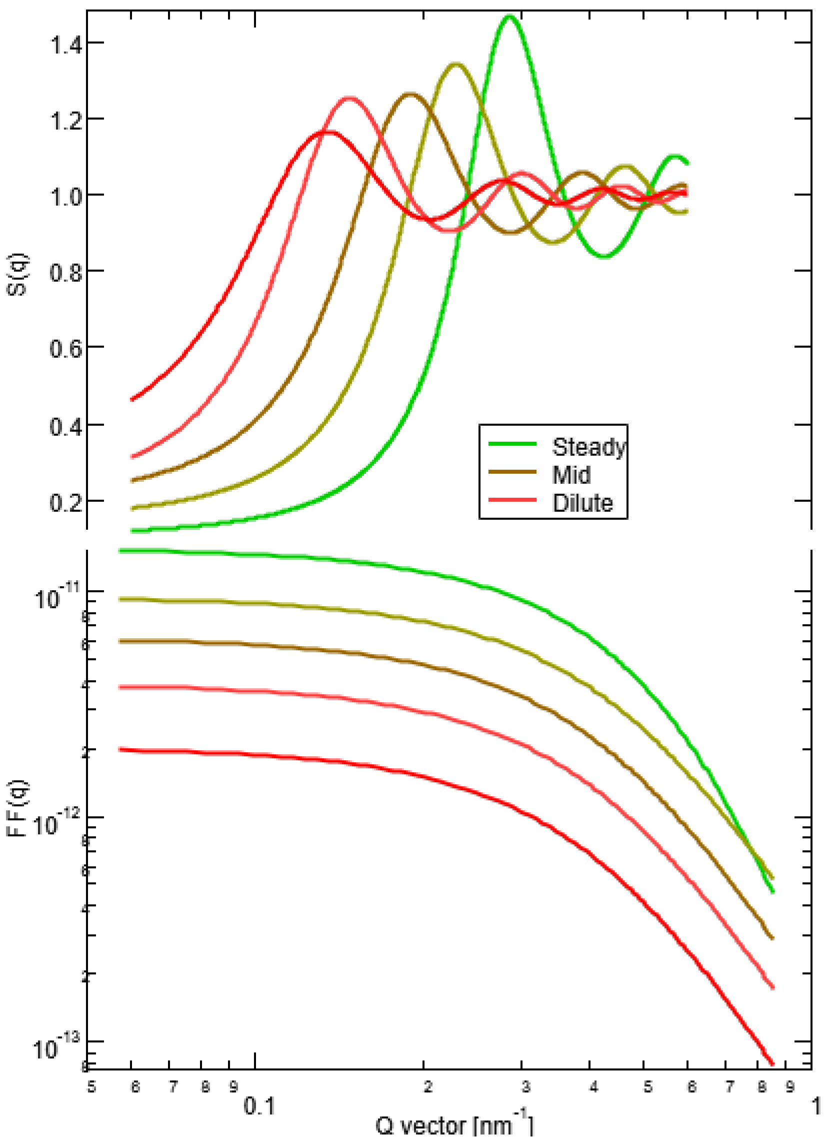

Supplementary figure 13.
Structure Factor (top) and Form Factor (bottom) components for figure 4c. All concentrations have similar Form Factor, but quite different Structure Fact

Supplementary Tables

Bead	W	PO	EO
W (Water)	25	54.37	26.05
PO (Propylene Oxide)	54.37	25	66.96
EO (Ethylene Oxide	26.05	66.96	25

Supplementary table 1.
Unitless conservative force parameters for DPD simulation

r_c [nm]	σ_c [nm]	r_{sh} [nm]	α [g/cm^2]	R_{HS} [nm]	ϕ_{HS} [%]
5.68(1)	3.88(4)	8.20(2)	508(1)	11.7(1)	18.0(1)

Supplementary table 2.
Table of structural fit parameters determined for 1% F127 through simultaneous fit as shown in Figure 2a. r_c is the average core radius, σ_c is the width of the polydisperse distribution, r_{sh} is the shell thickness, α is the areal density, R_{HS} is the hard-sphere radius, and ϕ_{HS} is the hard-sphere volume fraction. Details on how these are incorporated into our fit can be found in the methods section. Uncertainties are derived from built in Levenberg-Marquardt fitting routine in IGOR Pro 8.

Energy [eV]	$\left\|\Delta n_{c,sh}\right\|^2$	$\left\|\Delta n_{sh,sol}\right\|^2$	B	p	C
283	3.51e-6<br>(3.7e-8)	2.1e-8<br>(1.6e-9)	5.1e-18<br>(2e-19)	-3.1(1)	1.61e-13<br>(8e-16)
285	6.0e-6<br>(7.3e-7)	2e-8<br>(4e-8)	5.4e-18<br>(4e-19)	-3.1(1)	3.46e-13<br>(2e-15)
287	1.75e-5<br>(4.5e-7)	3e-8<br>(2e-8)	7e-19<br>(4e-19)	-3.2(2)	3.41e-13<br>(1.7e-15)
288.8	3.4e-5<br>(1.7e-6)	7e-8<br>(6e-8)	–	–	4.10e-13<br>(1.8e-15)
289.7	1.03e-5<br>(9.9e-7)	1.2e-7<br>(9e-8)	–	–	3.86e-13<br>(1.9e-15)

Supplementary table 3.
Energy dependent fit results from the same fit described in Supplementary table 2. B, p, and C represent the energy-dependent fit parameters in the power-law background as discussed in the methods. $I(q,E) = B(E) \cdot q^{p(E)} + C(E)$.

References

1. A. Guinier, G. Fournet, *Small-Angle Scattering of X-Rays* (John Wiley and Sons, New York, 1955).
2. D. J. Kinning, E. L. Thomas, Hard-Sphere Interactions Between Spherical Domains in Diblock Copolymers. *Macromolecules*. **17**, 1712–1718 (1984).
3. X. Song, S. Zhao, S. Fang, Y. Ma, M. Duan, Mesoscopic Simulations of Adsorption and Association of PEO-PPO-PEO Triblock Copolymers on a Hydrophobic Surface: From Mushroom Hemisphere to Rectangle Brush. *Langmuir*. **32**, 11375–11385 (2016).
